# Possible Involvement of Hydrosulfide in B_12_-Dependent Methyl Group Transfer

**DOI:** 10.3390/molecules22040582

**Published:** 2017-04-05

**Authors:** John I. Toohey

**Affiliations:** Cytoregulation Research, Elgin, ON K0G1E0, Canada; cytoreg@xplornet.com

**Keywords:** sulfane sulfur, hydrogen sulfide, methylation, radical S-adenosylmethionine methyl transferases, methionine synthase, cobalamin, viscose dialysis tubing, methionine auxotrophy, hypomethylation, dementia, Alzheimer’s disease

## Abstract

Evidence from several fields of investigation lead to the hypothesis that the sulfur atom is involved in vitamin B_12_-dependent methyl group transfer. To compile the evidence, it is necessary to briefly review the following fields: methylation, the new field of sulfane sulfur/hydrogen sulfide (S°/H_2_S), hydrosulfide derivatives of cobalamins, autoxidation of hydrosulfide radical, radical S-adenosylmethionine methyl transfer (RSMT), and methionine synthase (MS). Then, new reaction mechanisms for B_12_-dependent methyl group transfer are proposed; the mechanisms are facile and overcome difficulties that existed in previously-accepted mechanisms. Finally, the theory is applied to the effect of S°/H_2_S in nerve tissue involving the “hypomethylation theory” that was proposed 50 years ago to explain the neuropathology resulting from deficiency of vitamin B_12_ or folic acid. The conclusions are consistent with emerging evidence that sulfane sulfur/hydrogen sulfide may be beneficial in treating Alzheimer’s disease.

## 1. Introduction and Background

In recent years, sulfur in the form of sulfane sulfur (S°) or hydrogen sulfide (H_2_S) has been shown to have numerous regulatory effects in diverse biological systems [1,2]. The mechanisms proposed for its action include persulfidation of proteins particularly regulatory phosphatases, interaction with nitric oxide or cyclic guanosine monophosphate, and enhanced reduction capacity of glutathione (GSH) as the persulfide (GSSH) [3]. Evidence reviewed here suggests that this sulfur may function in another broad mechanism of regulation—B_12_-dependent methylation. The theory is especially applicable to nerve tissue where the sulfur factor and vitamin B_12_ appear to have similar effects both in their deficiencies and in supplementation.

## 2. Methylation, B_12_ Involvement, and Implication of Sulfur

**Scheme 1:**
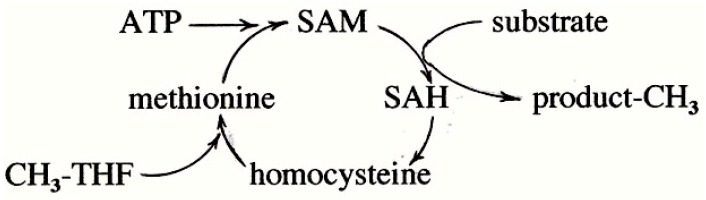
The Methylation Cycle. SAH, S-adenosylhomocyseine; ATP, adenosine triphoshate.

Biological compounds of enormous range are methylated to regulate various processes—DNA to regulate gene expression, RNA to regulate proteins synthesis, proteins to regulate activity, phospholipids to regulate lipid metabolism, as well as numerous small molecules (e.g., adrenaline). As shown in Scheme 1, the ultimate donor of methyl groups is usually S-adenosylmethionine (SAM). The end product of methyl group transfer from SAM is homocysteine which is remethylated by B_12_-dependent methyltetrahydrofolate (CH_3_-THF)-homocysteine methyl transferase also called methionine synthase (MS). The methyl group of CH_3_-THF originates in glycine, serine, or formate. A secondary cycle occurs in the liver where glycine is *N*-trimethylated directly by SAM to give betaine which can methylate homocysteine via betaine-homocysteine methyl transferase.

The methylation of oxygen and nitrogen atoms is relatively facile and is accomplished by simple S_N_2 reactions involving the transfer of the methyl cation, CH_3_^+^, from SAM to nucleophilic receptors. However, since the process is cyclic, all SAM-mediated methylation is indirectly dependent on the B_12_-dependent methylation of homocysteine. 

The methylation of carbon atoms is more difficult and involves radical SAM methyl transferases (RSMT) in which both the receptor carbon and the methyl cation are converted to radicals [4] (detailed in Section 6). The conversion of methyl cation to methyl radical occurs on an intermediate carrier. Based on the nature of the carrier, RSMT’s are classified into several classes. In Class A, the carrier comprises two conserved cysteine residues. In Class B, the carrier is a cobalamin (vitamin B_12_, 5,6-dimethylbenzimidazole cobalamin, Cbl) [5].

In mammals and bacteria, cobalamins are involved in methylation of homocysteine [6] and in Class B RSMT [5]. In archaea, methyl groups are transferred by corrinoids (cobamides in which the dimethylbenzimidazole of Cbl is replaced by other bases); in methanogenic archaea, the methyl group derived from carbon dioxide, acetate, or methanol is transferred to the sulfur atom of Coenzyme M and ultimately becomes methane; in acetogenic anaerobes, the methyl group derived from carbon dioxide is transferred from methylTHF to carbon monoxide dehydrogenase yielding acetate [7]. Plants do not have cobamides.

Sulfur has been implicated in at least two B_12_-dependent methylation systems. From purified MS, the cobalamin has been extracted as sulfitoCbl (detailed in Section 7.3). This was unexplainable prior to the discovery of the hydrosulfide derivative of Cbl (Section 4) and its oxidation to sulfite (Section 5). In the assay of B_12_-dependent RSMT, hydrosulfide is added to the assay mix in order to reconstitute the iron-sulfur cluster (Section 6) and it seems inevitable that the SHCbl derivative is formed. For the methanogenic and acetogenic systems, there is no evidence for sulfur on the Cbl but sodium sulfide is a component in the culture medium for the micro-organisms.

## 3. The New Field of Sulfane Sulfur/Hydrogen Sulfide

Sulfane sulfur (S°) and its 2-electron reduction product, hydrogen sulfide, have received a lot of attention in the literature in the last few years with implications of regulatory effects in many tissues and cells ([1,2,3] and the numerous references cited therein). Sulfane sulfur (technically thiosulfoxide sulfur or sulfur-bonded sulfur) is generated in mammals from cysteine by deamination giving mercaptopyruvate or from cysteine disulfides by C-S lyases such as cystathionine γ lyase (cystathionase) giving cysteine persulfide. Sulfane sulfur is transported on specialized transport proteins to conserved receptor sites on enzymes such as regulatory phosphatases. In many test systems, the agent has been added as NaHS which autoxidizes very quickly to S° [8,9]. A characteristic feature of sulfane sulfur is that it is effective at very low (nM) concentrations and must be maintained within very narrow concentration range. Supraoptimal amounts are toxic. A source of S° is required for in vitro proliferation of a group of cells with defective sulfur metabolism [10]. S° attaches to the sulfur of proteins to form persulfides (protein-S-SH) or polysulfides (pr-S-S_n_-S-pr) and it attaches to glutathione to give the persulfide (GSSH) which has a much greater reducing capacity than glutathione (GSH) (reviewed in [1]).

## 4. New Knowledge on Cobalamin Hydrosulfide

**Figure 1:**
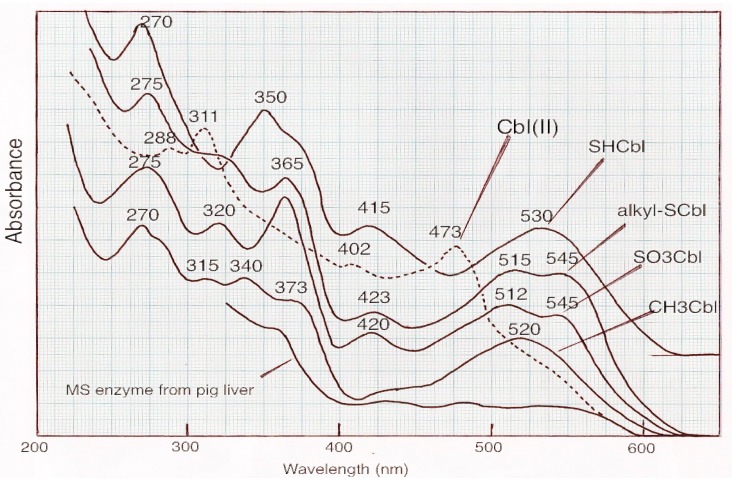
Absorption spectra of several cobalamins: methylCbl, sulfitoCbl, an alkylthioCbl (glutathionylCbl), hydrosulfideCbl, and Cbl(II) and an enzyme from pig liver [19].

Although the cyanide and other derivatives of cobalamin were characterized 60 years ago, the hydrosulfide derivative was not characterized until 1993 [11]. Hydrosulfide ion binds to the cobalt(III) in cobalamins [11,12], cobinamides [11,13], and cobaloximes [14]. The reaction with H_2_OCbl initially gives the purple derivative SHCbl(III) that is similar to the derivatives given by thiols (λ_max_ 270, 350, 415 and 530 nm) [15]. However, over several minutes, there is an internal electron shift from the sulfur atom to the cobalt atom to yield a stable structure represented by Co(II)H:S::•. When the reaction is carried out at a HS^−^/Cbl ratio of 2 and at pH 8 under aerobic conditions, the final product is ruby-brown, stable to light and to air, paramagnetic, and has an absorption spectrum quite different from that of Cbl(II) [11] (Figure 1). The same ruby-brown product is obtained when fully-reduced cobalamin, Cbl(I), is mixed with elemental sulfur, indicating that there is a series of electron shifts in equilibrium as shown in Equation (1):

[Co(III)] H:S:::^−^ ⇄ [Co(II)] H:S::• ⇄ [Co(I)]:S::^(^°^)^ (1)

Since the product is paramagnetic, it appears that the predominant form is the middle one containing the hydrosulfide radical. Under the conditions described above (pH 8, aerobic) there is evidence that this radical stabilizes by forming the dimer Cbl(II)-S-S-Cbl(II) [11,12]. As with thiols [15], Cbl is sensitive to reduction by excess hydrosulfide to give free Cbl(II). In a recent study [12], Salnikov et al. determined the rate constants for this reduction using a huge excess of hydrosulfide. They used a molar ratio HS**^−^/**Cbl of 1000 and the product observed was mainly Cbl(II); however, at pH 4.5 under anaerobic conditions, they observed an absorption spectrum which suggests a mixture of Cbl(II) and the ruby-brown product.

## 5. Oxidation of Hydrosulfide Radical

In studying the reaction of Cbl with H_2_S, Salnikov et al. found that, when the aqueous solution was exposed to bubbling air for 10 minute at pH 7 to 8, the SHCbl product was converted to sulfitoCbl in high yield [12]. The oxidation chemistry of hydrosulfide radical was studied by Zhu et al. [16]. Hydrosulfide ion, HS:^−^, is not exceptionally reactive but hydrosulfide radical, HS•, is highly reactive with oxygen, the end product being sulfite. The sequence of reactions is shown in Equations (2)–(5). 

HS• → S•^−^ + H**^+^** (2)

S•^−^ + O_2_ → SOO•^−^ (3)

SOO•^−^ + O_2_ → SO_2_ + O_2_•^−^ (4)

SO_2_ + H_2_O → H^+^ + HSO_3_^−^ (5)

The HS• radical dissociates at pH ~ 7 to give sulfide radical S•^−^. This radical reacts with oxygen to give the peroxy radical SOO•^−^ which reacts with another oxygen to give sulfur dioxide and superoxide. Sulfur dioxide reacts with water at neutral pH to form sulfite ion.

## 6. The Mechanism of B_12_-Dependent Radical SAM Methylation

**Scheme 2:**
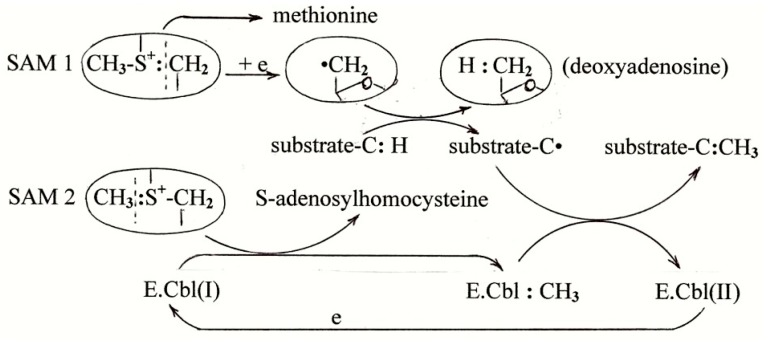
Radical SAM methyl transfer. Only the structure around the sulfonium ion of SAM is shown.

RSMT enzymes have an iron-sulfur cluster [4Fe-4S] which self-constructs on three cysteine residues of a conserved motif, CxxxCxxC [4]. Two molecules of SAM are consumed for each methylation as shown in Scheme 2.

The Fe-S cluster donates an electron to convert one SAM into methionine and 5′-deoxyadenosine radical, dAdo•. This radical abstracts a hydrogen atom (one proton and one electron) from the methyl receptor carbon and ends up as 5′-deoxyadenosine. A second molecule of SAM provides the methyl group and ends up as S-adenosylhomocysteine. The methyl group attaches to the carrier as the cation, CH_3_^+^, and leaves as the methyl radical, •CH_3_, which then joins with the substrate radical to give the methylated product. The conversion of the methyl cation to the radical consumes an electron which ends up in the product so that a reducing system is needed to complete the cycle. 

As stated above, Class B RSMT’s contain Cbl as the methyl group carrier [5]. During the purification of these enzymes, the Fe-S cluster becomes disrupted and has to be reconstituted during the transmethylase assay by adding NaHS and an iron salt. Since both Cbl and HS**^−^** ion are present, the latter in huge excess, it is likely that the Cbl is fully converted to the hydrosulfide derivative. As discussed below, the cobalt-sulfur unit provides an excellent means for converting the methyl cation to the methyl radical.

## 7. Methionine Synthesis

### 7.1. The Accepted Mechanism of Methionine Synthesis

**Scheme 3:**
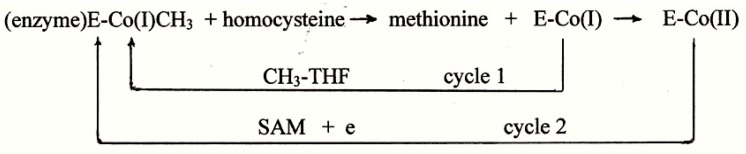
The accepted mechanism of B_12_-dependent methionine synthase.

The system requires anaerobic conditions and involves tightly-bound cobalamin, a powerful reducing system, and two methyl group donors, SAM and methyltetrahydrofolate (CH_3_-THF) [6]. As shown in Scheme 3, enzyme-bound CH_3_Cbl is believed to be the ultimate donor of the methyl group to homocysteine. The methyl group is transferred as the cation CH_3_^+^ leaving the cobalt in the form of Co(I) which can be remethylated by CH_3_THF (cycle 1). The highly-reduced Co(I) undergoes occasional oxidation to Co(II) which requires a strong reductant and SAM for remethylation (cycle 2). Certain features of this system have been persistently difficult to explain as outlined below.

*Reducing System:* Despite the fact that electrons are not consumed in the reaction and the reaction is carried out in strict anaerobic conditions, a powerful reducing system is still required. Many reducing systems have been used or implicated in MS: FMNH_2_, FAD-platinum-H_2_, FAD and NADPH, mercaptoethanol or dithiothreitol plus OHCbl, titanium(III) citrate, coupled flavoproteins R and F in *Escherichia coli*, cytochrome b_5_ with a cytochrome reductase, and methionine synthase reductase in mammals. None of these has an E′o value that approaches the value required to reduce Cbl(II) to Cbl(I), −0.7 volts.

*SAM Requirement:* The requirement for two methyl donors (SAM in particular) has always been difficult to explain. The reported effects of adding SAM to the assay mixture have been extremely variable. For the highly-purified enzyme, some reports describe an absolute requirement for SAM, for example [17,18], while other reports describe only partial stimulation of activity by SAM in preparations from human placenta, pig kidney, or bovine brain, for example [19], in which SAM addition caused only ~30% to 45% stimulation. Most studies determined the SAM requirement only at the end of lengthy purification procedures but, in one study, Rudiger and Jaenicke followed the effect of SAM at each stage of purification [20]. They found that SAM was not required in the crude extract and that its requirement increased progressively at each step reaching 99% requirement after the last step. This is consistent with findings of this author that SAM has no effect on MS specific activity in crude extracts of cells grown in culture [21]. This suggests that a factor other than SAM is lost during purification and that, in the absence of that factor, SAM rather than CH_3_THF is required as the source of the methyl group. Evidence presented here suggests that the lost factor is a sulfur atom.

### 7.2. Viscose Dialysis Membrane

The details of the reports on MS show that the purification procedures for the SAM-dependent preparations involved little or no dialysis while the purification of largely SAM-independent preparations involved numerous dialysis steps with the enzyme enclosed in dialysis membrane for as much as 90 h. As discussed below, prolonged exposure to viscose dialysis membrane may have introduced sulfur onto the cobalt negating the requirement for SAM. 

**Scheme 4:**
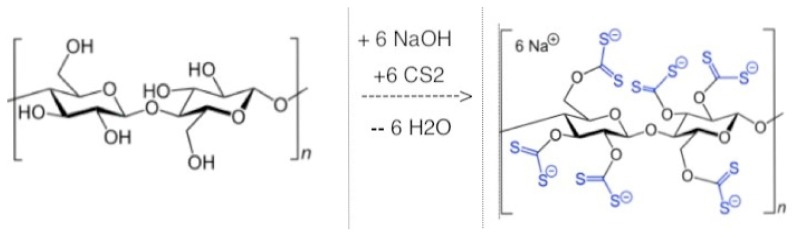
The reaction of carbon disulfide with cellulose in the viscose process [22].

The viscose process involves dissolving cellulose in strong alkali and treating with carbon disulfide, as shown in Scheme 4. Much of the CS_2_ is removed with sulfuric acid in a later step but dialysis membrane, even today, retains up to 0.3% sulfur (ThermoFisher Scientific Catalog, ThermoFisher Scientific, Waltham, MA, USA).

In the 1960–1970s, suppliers provided instructions suggesting exhaustive boiling in water to remove the sulfur; now sulfur-removal kits are available. The Methods sections of papers from the 1960s do not indicate how dialysis tubing was prepared for use; in any case, it is highly likely that sulfur would continue to be released during dialysis. Even traces of sulfur could have significant effects since sulfane sulfur is biologically active at low nM concentrations. This is demonstrated by the fact that, when sulfane sulfur-dependent cell lines were cultured in Marburg vessels (culture vessels with two chambers separated by dialysis membrane), the cells grew well without addition of any other source of sulfane sulfur [10]. This happened despite the fact that the dialysis membrane had been exhaustively boiled. It seems obvious that the dialysis membrane provided the S° for the S°-dependent cells.

Introduction of sulfur from dialysis membrane is not limited to the cell culture example but may apply to enzymes which have polysulfide links. For example, copper-zinc superoxide dismutase has been shown to have a polysulfide link of variable length up to seven atoms [23]. The polysulfide link appears to be natural but the possibility exists that some of this sulfur may have been introduced from dialysis tubing during purification. Similarly, the use of “dialyzed fetal calf serum” could mask a requirement for S° in certain cultured cells.

### 7.3. The Nature of the Cobalamin in Methionine Synthase

Highly purified preparations of the enzyme are pink or salmon colored. Absorption spectra were used to try to identify the nature of the cobalamin with variable results. Various purified enzymes revealed spectra characteristic of OHCbl [17], Cbl(II) [24,25] or methylCbl [20]. This type of evidence has drawbacks: (a) the spectra are crude when the sample is not highly purified; (b) the more the manipulation and the higher the purity, the more chance of stripping off labile components; and (c) many cobalamin compounds have similar spectra. This is demonstrated in Figure 1 which shows the absorption spectra of several cobalamin derivatives. It is apparent from the spectra shown that it would be difficult to distinguish most of these derivatives on the basis of the absorption properties. 

In 1961, Takeyama and Buchanan extracted the cobalamin from the enzyme purified from *Escherichia coli* using hot ethanol and published a spectrum now known to be identical to that of sulfitoCbl. They did not mention sulfito-Cbl and they did not establish an identity [26]. In 1968, Weissbach’s group repeated this experiment and published an identical spectrum [27]. They used chromatography and electrophoresis to compare the mobility of the unknown with that of several cobalamins and showed that it moves with sulfitoCbl. It is likely that the enzyme containing sulfitoCbl is inactive in the enzyme assay. 

In many laboratories, it was found advantageous to carry out the entire purification procedure of MS in the presence of mM concentrations of a thiol such as glutathione [18], mercaptoethanol [20], or homocysteine [19]. If, as proposed here, the Cbl was originally in the SH form, it is likely that the thiol helped to prevent oxidation of SHCbl to sulfitoCbl. It is interesting that no thiol was used in the preparations in which sulfitoCbl was identified. Even when protected from oxidation by the presence of thiols, it is uncertain whether SHCbl could remain intact during lengthy purification procedures but data on this are not available.

### 7.4. Excess Vitamin B_12_ Added to Cell Culture Greatly Increases MS Specific Activity

In 1969, Mangum et al. reported the effect of adding excess vitamin B_12_ (0.3 μM) to the medium of several cell lines cultured in Minimum Essential Medium (normal B_12_ content ~ 1 nM contributed by the 10% calf serum). The specific activity of MS in the crude cell extracts increased by as much as 30-fold [28]. The cells included normal and malignant human and hamster cell lines. The effect occurred within a few hours and was not due to conversion of apoenzyme to holoenzyme and was not affected by adding homocysteine along with vitamin B_12_. In the present context, it is possible to explain the effect of excess Cbl on MS activity in cultured cells when the following facts are taken into consideration:

(a) Animal cells grown in vitro secrete cysteine and homocysteine into the medium [29]
(b) Cobalamin compounds are exceptionally effective in catalyzing the autoxidation of sulfhydryl compounds to disulfides: cys-SH + R-SH + ½O_2_ → cys-S-S-R + H_2_O [30]
(c) Cysteine disulfides are degraded by C-S lyases to give persulfides according the following equation: cys-S-S-R → NH_3_ + pyruvate + cy-S-S-H [1].

The sulfur, either as S° or H_2_S, could attach to the Cbl of MS in the cells greatly increasing the efficiency of the enzyme. The extracts from cultured cells were not subjected to purification procedures so that the sulfur would not be lost from the Cbl cofactor as it is during large scale enzyme preparations.

### 7.5. Methionine Auxotrophy

In 1974, somewhat limited evidence gave rise to the theory that cancer cells but not normal cells cultured in vitro are dependent on methionine in the medium, i.e., that normal cells but not cancer cells can proliferate if methionine is replaced by homocysteine [31]. In the following 30 years, scores of papers were published exploring every aspect of this subject and there was contradictory evidence on almost every point. No satisfactory explanation had emerged when the subject was reviewed in 2003 [32] and in 2012 [33]. Although the phenomenon is not restricted to cancer cells, it is certain that some cell lines are methionine-dependent.

In 2009, Watkins et al. reported the significant finding that a methionine-dependent human melanoma cell line (MeWoLC1) had a defect in the cblC locus [34,35]. This gene codes for the chaperone protein MMACHC which is responsible for assembling the cobalamin coenzymes in situ. Thus, external sources of Cbl (e.g., CNCbl, methylCbl) are not incorporated directly into enzymes [36] but are degraded to hydroxyCbl before incorporation into Cbl-containing enzymes by the chaperone protein [37]. According to the current theory, it seems possible that this protein incorporates a sulfur atom into the Cbl cofactor as it is assembled in MS. A defect in the cblC gene is only one defect of many that could impair this sulfur incorporation. Other defects that could impair the process include well-known defects in sulfane sulfur generation or S°-carrying proteins [1]. According to the theory proposed here, impaired incorporation of sulfur into the Cbl of MS would result in decreased methionine synthesis and ultimately to deficiency of SAM and impaired methylation. This is discussed further in Section 8.4.

## 8. Putting the Information Together

Evidence cited above suggests a role of the sulfur atom in B_12_-dependent methyl group transfer. This raises the question of the mechanism. Two mechanisms need to be considered;

(a) sulfur atom may facilitate the formation of MeCbl which acts as the methyl group carrier or
(b) sulfur atom may bind to the cobalt and the cobalt-sulfur unit acts as the methyl group carrier.

### 8.1. Possible Catalytic Role of Sulfur in Formation of MeCbl

B_12_-dependent methylation can be considered to occur in two stages; (a) formation of MeCbl and (b) transfer of the methyl group to the acceptor substrate. It is well-known that, in chemical systems, homocysteine removes the methyl group from CH_3_Cbl non-enzymatically (for a key to this literature, see [38]). Therefore, the main energy barrier is not the transfer of the group from MeCbl to the acceptor but in the formation of MeCbl. 

**Scheme 5:**
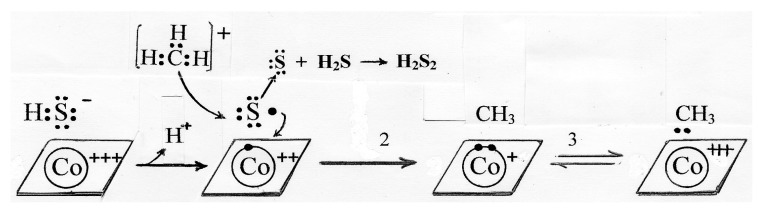
Proposed mechanism for the synthesis of MeCbl by the method of Dolphin & Johnson.

In the formation of MeCbl, both chemical and enzymatic, the methyl group is provided in the form of the methyl cation, CH_3_^+^. Electromagnetic properties prevent this cation from approaching the cobalt in Cbl(III)or Cbl(II). Therefore, the conventional chemical method of preparing MeCbl is to reduce the Cbl to the green Cbl(I) stage and then to add methyl iodide [39]. In 1963, Dolphin and Johnson in a brief communication, described the preparation of crystalline MeCbl in high yield in a simple system involving hydrosulfide [40]. They combined OHCbl and NaHS to give first a purple product and then a brown product resembling Cbl((II); addition of methyl iodide yielded MeCbl in 90% yield. There was no evidence of Cbl(I) involvement. This unconventional and largely-overlooked method needs confirmation and further investigation. A tentative interpretation is shown in Scheme 5. In the SH derivative of Cbl, an internal electron shift occurs from the sulfur atom to the cobalt. This converts the SH^−^ ion to the radical which is known to dissociate at pH ~ 7 [16] so that the rest of the process involves sulfide radical or sulfur atom. It appears likely that the methyl cation displaces the sulfur atom as S° leaving a second electron on the cobalt. Alternatively, the second electron may be provided by the excess NaHS.

This mechanism is supported by two findings reported in the literature. The first is data obtained with purified MS [41]. The enzyme had been exposed to six dialysis steps (totaling ~ 90 h), the final step being an 8 h dialysis. Therefore this enzyme was likely to have been saturated with sulfane sulfur from the viscose tubing both on the Cbl and on susceptible cysteine residues. The enzyme in the presence of the reducing system (DTT), had the spectrum of Cbl(II) but addition of SAM changed the spectrum to that of Cbl(I) [41]. The system contained homocysteine, which, as stated above, strips the methyl group from the MeCbl leaving Cbl(I). Therefore, the rate-limiting step was the formation of MeCbl. Since SAM does not methylate Cbl(II), the only explanation for the observations appears to be that the methylation of Cbl was facilitated by the presence of sulfur directly comparable to the system of Dolphin and Johnson. The sulfur could have been on the Cbl or carried as persulfide on cysteine residues and released as H_2_S by the DTT. 

The second evidence supporting a role of sulfur in MeCbl formation comes from findings of Berteau et al. in studying a B_12_-dependent RSMT system which methylates tryptophane during the synthesis of the antibiotic thiostrepton [42]. When the authors combined OHCbl, DTT, SAM, and enzyme that had been reconstituted with NaHS and iron (the enzyme was not freed of excess Na HS), they detected formation of MeCbl. They found no involvement of Cbl(I). There was an equivalent conversion of the SAM to SAH indicating stoichiometric methylation of Cbl by SAM in the presence of hydrosulfide ion. The enzyme appeared to be required.

### 8.2. Sulfur as Part of the Carrier in Methionine Synthase

**Scheme 6:**
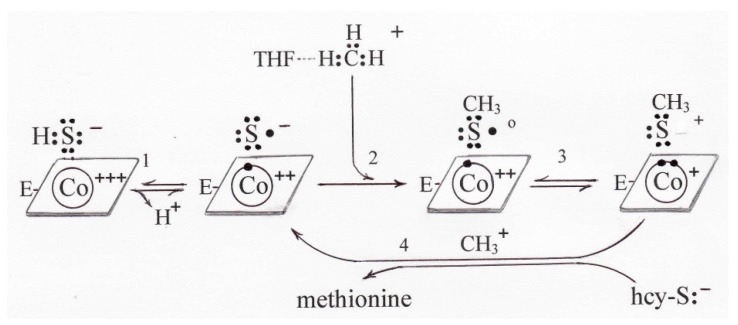
A possible mechanism for methionine synthase involving sulfide radical. The mechanism shows the involvement of Cbl(I) since there is spectrophotometric evidence for the occurrence of Cbl(I) in the accepted mechanism [41]. However, as described below, the “accepted” mechanism involving Cbl(I) may be an in vitro artifact in which case, Cbl(I) need not be included in Scheme 6.

The second alternative is that the sulfur remains on the cobalt and the two together form a methyl-carrying unit. One possible mechanism for MS is shown in Scheme 6. Step 1 shows one electron moving from the sulfur to the cobalt and dissociation of the H^+^ ion to give sulfide radical. Step 2 shows the methyl cation moving to the negative sulfur atom. Step 3 shows a resonance between Co(II)S·CH_3_ and Co(I)S^+^·CH_3_ which would facilitate transfer of the CH_3_^+^ cation to the negative sulfur on homocysteine (step 4).

The mechanism proposed for methionine synthesis in Scheme 6 does not, in any way, diminish the validity of the mechanism in Scheme 3. Both mechanisms may be valid but under different circumstances. The mechanism in Scheme 3 undoubtedly applies to the highly-purified enzyme which has lost the sulfur atom. The mechanism in Scheme 6 is proposed to apply in the following conditions:

- crude tissue extracts which have not been manipulated in such a way that the sulfur is lost,
- purified enzyme which has been protected by thiols during purification,
- purified enzyme that has been exposed for prolonged periods to dialysis membrane, and
- crude extracts of cells which have been cultured in the presence of ~1 μM vitamin B_12_ under conditions that can generate S° (as in reference [28]).

The evidence suggests that the sulfur-containing mechanism (Scheme 6) is about 30 times more efficient that the mechanism in Scheme 3 [28] and that many systems described in the literature were comprised of a mixture of the two mechanisms.

### 8.3. Sulfur as Part of the Carrier in RSMT

**Scheme 7:**
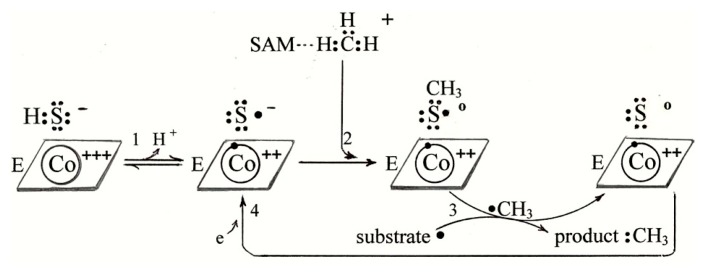
A mechanism for hydrosulfide involvement in B_12_-dependent RSMT.

A similar mechanism may apply to involvement of sulfide in the B_12_-dependent RSMT’s. Since most assay systems contain both Cbl and hydrosulfide ion, it is likely that the Cbl is fully in the SHCbl form. Again, it is possible to draw different effective mechanisms; the mechanism shown in Scheme 7 does not involve Cbl(I) since the evidence shows the presence of Cbl(II) only [42]. Step 1 is the same as step 1 in Scheme 5 and Scheme 6 (one electron moves from the sulfur to the cobalt and the resulting hydrosulfide radical dissociates). In step 2, the positive methyl cation moves from SAM to the negative sulfur radical. In step 3, the methyl group moves as a radical to the substrate radical. This removes one electron which ends up in the product. Therefore, step 4 is necessary to replace this electron.

### 8.4. The Hypomethylation Theory and Dementia

**Scheme 8:**
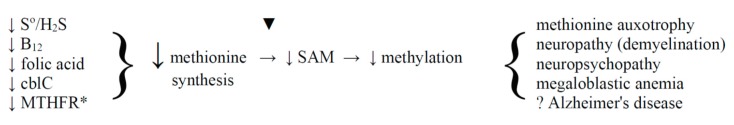
The Hypomethylation Theory. The symbol ↓ represents “decreased”. * Congenital deficiency of methylenetetrahydrofolate reductase (MTHFR) interferes with folic acid metabolism and causes hyperhomocsyteinemia and decreased blood levels of methionine and SAM [48]. **▼** Congenital deficiency of methionine adenosyl transferase with hypermethioninemia causes demyelination in the brain [49].

As discussed in Section 7.5, decreased availability of S°/H_2_S could lead to decreased methionine synthesis, decreased SAM availability, and ultimately to hypomethylation of essential sites. With cells in culture, this would account for methionine auxotrophy but, in the intact animal, this has broader implications especially relevant to nerve tissue. This association calls to mind the theory of the 1990’s that the neuropathology of vitamin B_12_ deficiency is due to hypomethylation of proteins in nerve tissue—a theory for which there was a considerable body of evidence (not reviewed here but see [43]). Folic acid is co-involved with vitamin B_12_ in the methylation cycle and folic acid deficiency causes the same neuropsychiatric symptoms as vitamin B_12_ deficiency [44]. Numerous recent reports suggest that dementia may be prevented or treated with supplemental vitamin B_12_ (see [45]), folic acid [46], or S-adenosylmethionine [47]. The results with folic acid suggest an epigenetic mechanism in which gene methylation prevents overproduction of the amyloid-forming proteins [46]. The theory of hypomethylation is summarized in Scheme 8 and expanded to include the sulfur species and methionine auxotrophy. 

Aside from the requirement for vitamin B_12_ and folic acid to maintain nerve function, there is emerging evidence that S°/H_2_S is also required, for example the possible curative effects of this sulfur in dementia [50,51]. Brain and nerve tissue have a unique susceptibility to S°/H_2_S deficiency because they are semi-isolated from the rest of the body. Brain tissue depends on MS for recycling homocysteine because the betaine-homocysteine methyl transferase does not occur in the brain. It is likely that the cysteine deaminase–mercaptopyruvate sulfur transferase (CT-MST) pathway is the only source of S°/H_2_S in the brain since the transsulfuration enzymes have doubtful status in the brain. Cystathionine γ-lyase has been reported to be undetectable in brain tissue and to be completely absent in the embryo (summarized in [52]). Cystathionine β-synthase has been shown to be expressed in brain but it is unlikely to be significant source of S°/H_2_S in brain because its Km for cysteine (36 mM) is much greater than the Km for the conventional substrate serine (~3 mM) [53] and far beyond the physiological level of cyst(e)ine in cerebrospinal fluid (1.1 ± 0.4 μM) [54]. Furthermore, S°/H_2_S generated elsewhere in the body cannot enter nerve tissue because it is bonded to carrier proteins that cannot cross the blood-brain barrier. Cysteine transaminase activity occurs in brain tissue at activity fractionally lower than in liver (1.42 versus 3.78 μmol H_2_S/min/gm of tissue [53]). MST has been found in all tissues examined [52] and the CT-MST system has been shown to operate in brain tissue [55] and peripheral nerves [56].

Aside from the evidence that the CT-MST pathway is the main or only source of S°/H_2_S in nerve tissue, there is other evidence relating its deficiency to the neuropsychopathy as shown in Scheme 8. Thus, Nagahara et al. generated an MST knockout mouse [57] which showed neuromental dysfunction comparable to the neuropsychiatric abnormalities which are the early signs of vitamin B_12_ or folic acid deficiency. Also, a recent series of reports showed curative effects of a sulfur donor in mouse models of Alzheimer’s disease (see [58]). The sulfur donor, triphenylphosphonium-(CH_2_)_9_-C(O)-*O*-phenyldithiolethione, called AP39, is an ester that is likely to be lipid soluble and to pass through the blood-brain barrier and to supply supplemental sulfur to the brains of the mice.

In summary, it is proposed that three factors are essential for maintaining methylation and function in nerve tissue—vitamin B_12_, folic acid, and S°/H_2_S. It is not presently possible to define the mechanism by which methylation protects nerve function. Possibilities include (a) methylation of homocysteine would remove its toxic effects; (b) methylation of proteins in nerve tissue may prevent their aggregation as amyloid; and (c) methylation of genes may suppress synthesis of the amyloidogenic proteins (as suggested in [46]).

## 9. Conclusions and Perspective

Many lines of evidence (previously difficult to explain in themselves) converge to support the hypothesis that the sulfur atom is involved in B_12_-dependent methylation. The evidence is particularly strong for the much-studied MS being chemical, enzymatic, and from cell culture:

− isolation of sulfitoCbl from the air-exposed purified enzyme,
− decreased need for SAM in much-dialyzed enzyme (given that viscose membrane releases S°),
− 30-fold increase in activity in cells cultured in conditions which favor S° formation, and
− methionine auxotrophy in cells with defective sulfur metabolism.

It is likely that, during long purification procedures, the sulfur atom is lost (or oxidized to sulfite). This loss may force the enzyme into an artificial and inefficient mode of action. The theory might be tested by adding HS^−^ ion to the MS assay system. The theory predicts that this will eliminate the need for SAM and greatly increase the specific activity (the latter based on the findings of Mangum and North [28]). Adding HS^−^ ion may also permit activity in aerobic conditions; however, in the presence of air, it may be necessary to also include superoxide dismutase and catalase since the air oxidation of hydrosulfide to sulfite is known to generate damaging reactive oxygen species. The superoxide dismutase would be needed to convert the hydroxyl radical to H_2_O_2_ and catalase would be needed to destroy the H_2_O_2_ .This is a system which occurs naturally in tissues.

It will be more difficult to test the theory in the B_12_-dependent RSMT systems because hydrosulfide ion is already added to the assay systems. Since Cbl and HS^−^ ion are both present in these assay systems, it seems inevitable that the Cbl on RSMT’s is present in the form of SHCbl but it remains to be determined whether the sulfur is involved in the methyl transfer and, if not, how it is displaced by the methyl group. 

The diverse physiological effects of sulfane sulfur/hydrogen sulfide suggest that it acts in a generalized way through other regulators. The precise target in any system is not yet known but the sulfur has been proposed to act in the following regulatory systems: the nitric oxide system, the cyclic GMP system, the phosphatase system (by persulfidation of the phosphatase enzymes), and the redox system (by forming persulfides which have much greater reducing capacity than thiols, e.g., G-S-S-H > GSH). This review suggests its participation in another regulatory system of broad scope—B_12_-dependent methylation. Possibly all of the mechanisms apply in various systems but the study of mechanism of action of this form of sulfur is only just beginning.
